# P-516. Statewide Targeted Congenital CMV Testing in Newborns Who Underwent Hearing Screening in Connecticut: A Nine-Year Longitudinal Study

**DOI:** 10.1093/ofid/ofaf695.731

**Published:** 2026-01-11

**Authors:** Ashley Howard, Ryan Manthey, John Lamb, Ian Michelow

**Affiliations:** University of Connecticut, Hartford, CT; Connecticut Childrens, Hartford, Connecticut; CT Department of Public Health, Hartford, Connecticut; University of Connecticut, Hartford, CT

## Abstract

**Background:**

Congenital cytomegalovirus infection (cCMV) is the leading acquired cause of sensorineural hearing loss worldwide, occurring in up to 50% of affected newborns if symptomatic at birth, and in up to 15% if clinically inapparent. Approximately 0.4% of newborns in the United States are infected with CMV. In 2016, Connecticut (CT) implemented House Bill 5525, "An Act Concerning Cytomegalovirus", mandating targeted cCMV screening for all infants who fail the newborn hearing screen. The goal of this study was to analyze the impact of this bill.

**Table 1.**
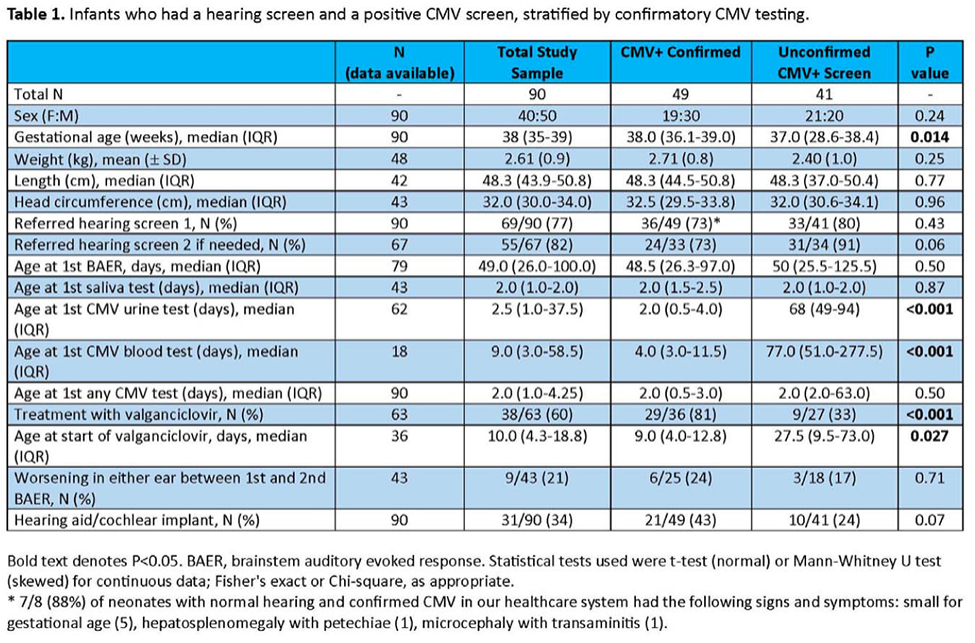
Infants who had a hearing screen and a positive CMV screen, stratified by confirmatory CMV testing.

**Figure 1.**
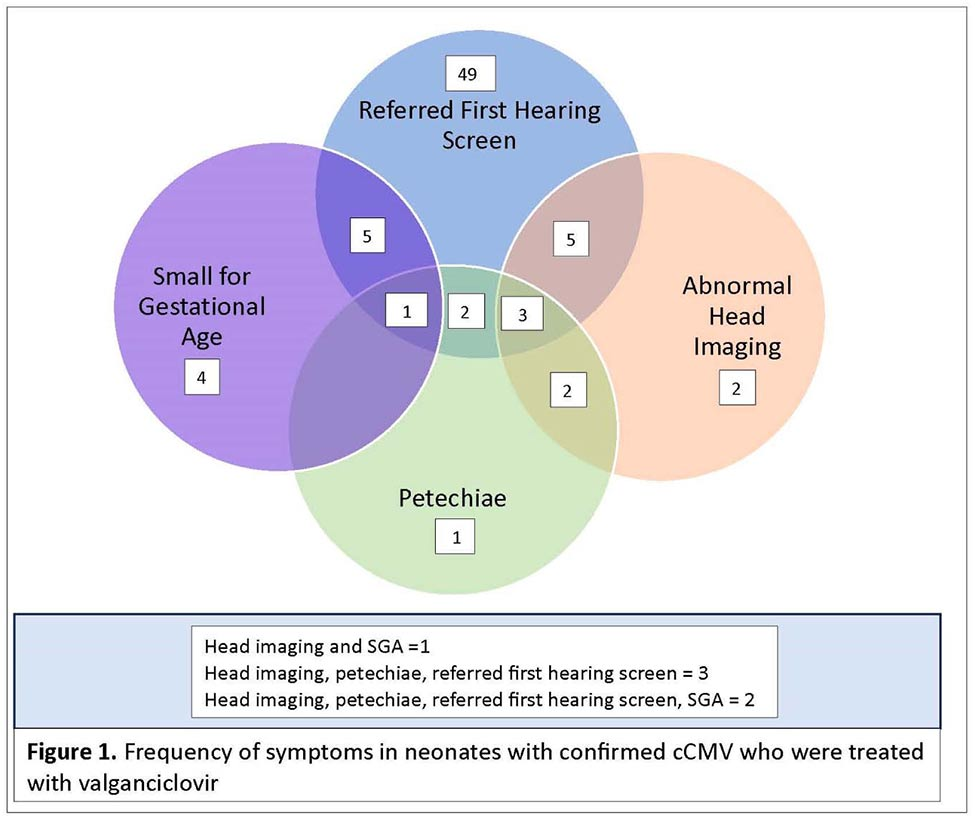
Frequency of symptoms in neonates with confirmed cCMV who were treated with valganciclovir.

**Methods:**

We conducted a retrospective longitudinal study of consecutive infants born in CT who failed their newborn hearing screen and underwent CMV testing from 1/1/2016 to 4/28/2025. Infants were identified through a state database with further detailed characterization if care was received in our healthcare system, which receives > 50% of referrals for cCMV in the state. Diagnosis of cCMV was considered confirmed if blood or urine PCR tests were positive < 21 days of life. Infants with a positive saliva PCR result but no confirmatory tests, and those with blood or urine PCR results > 21 days of life were considered unconfirmed. Descriptive and comparative statistics were performed. A two-tailed P value < 0.05 was considered statistically significant.

**Results:**

Overall, 54 of 3839 (1.4%) infants who failed their newborn hearing screen were confirmed to have cCMV. In a separate detailed analysis from 1/1/2016-12/31/2024, 90 infants screened positive for cCMV. Of these, 39 of 49 (80%) confirmed cases received care within our healthcare system. Table 1 shows the characteristics of the confirmed and unconfirmed cases.

Figure 1 illustrates the frequency of symptoms in confirmed cases who were treated with valganciclovir.

**Conclusion:**

We identified premature infants as being at risk for delayed hearing screening and confirmation of cCMV. Almost one-half of confirmed cases required hearing aids or cochlear implants, confirming the serious long-term sequelae caused by cCMV. Universal newborn CMV screening will facilitate earlier linkage to care and initiation of definitive therapy to optimize developmental and hearing outcomes.

**Disclosures:**

All Authors: No reported disclosures

